# Structure and dynamics of rotary V_1_ motor

**DOI:** 10.1007/s00018-018-2758-3

**Published:** 2018-01-31

**Authors:** Hiroshi Ueno, Kano Suzuki, Takeshi Murata

**Affiliations:** 1 Department of Applied Chemistry, Graduate School of Engineering, University of Tokyo, Tokyo, 113-8656 Japan; 2 Department of Chemistry, Graduate School of Science, Chiba University, Chiba, 263-8522 Japan; 3 JST, PRESTO, Chiba, 263-8522 Japan

**Keywords:** Rotary catalysis, Ion pump, *Enterococcus hirae*, Crystal structure, Single-molecule technique

## Abstract

Rotary ATPases are unique rotary molecular motors that function as energy conversion machines. Among all known rotary ATPases, F_1_-ATPase is the best characterized rotary molecular motor. There are many high-resolution crystal structures and the rotation dynamics have been investigated in detail by extensive single-molecule studies. In contrast, knowledge on the structure and rotation dynamics of V_1_-ATPase, another rotary ATPase, has been limited. However, recent high-resolution structural studies and single-molecule studies on V_1_-ATPase have provided new insights on how the catalytic sites in this molecular motor change its conformation during rotation driven by ATP hydrolysis. In this review, we summarize recent information on the structural features and rotary dynamics of V_1_-ATPase revealed from structural and single-molecule approaches and discuss the possible chemomechanical coupling scheme of V_1_-ATPase with a focus on differences between rotary molecular motors.

## Introduction

All living cells use a chemical fuel called adenosine triphosphate (ATP) to maintain the functions required for life, such as protein biosynthesis, muscle contraction, and brain activity. Therefore, ATP is often referred to as the energy currency for the cell. Under aerobic conditions, the majority of ATP is produced by the F-type ATP synthase [1]. F-type ATP synthases (also known as F-ATPases) are ubiquitous rotary motor enzymes found in the inner membrane of mitochondria, the thylakoid membrane of chloroplasts, and the plasma membranes of bacteria. They catalyze ATP synthesis from ADP and inorganic phosphate using the energy of ion (proton or sodium) translocation caused by transmembrane electrochemical potential (proton or sodium ions) and, when operating in reverse, they also generate an electrochemical potential difference of ions using the energy released by ATP hydrolysis [2–4].

**Fig. 1:**
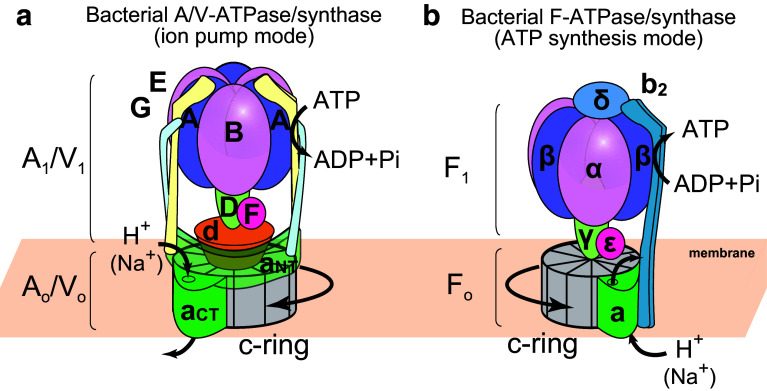
Schematic illustrations of bacterial rotary ATPases. **a** A/V-type ATPases/synthases are found in Archeae and some bacterial taxa. They are composed of a soluble catalytic core A_1_/V_1_ motor (A_3_B_3_DF) and a membrane integral A_o_/V_o_ motor (ac_*n*_d; where *n* is the copy number of the c subunits). The A_1_/V_1_ and A_o_/V_o_ motors are connected by the two peripheral stalks (EG). The N- and C-terminal domains of a subunit are shown as a_NT_ and a_CT_, respectively. **b** Bacterial F-type ATPases/synthases are structurally different from bacterial A/V-type ATPases/synthases. The F_1_ (α_3_β_3_γδε) and F_o_ motors (ab_2_c_*n*_) are connected by only one peripheral stalk (b_2_)

Eukaryotic vacuolar-type ATPases (V-ATPases) are also rotary motor enzymes; they are evolutionarily and functionally related to F-type ATP synthases [5, 6]. They function in reverse of F-type ATP synthases, that is, they transport ions (protons or sodium ions) across the membrane using the energy derived from ATP hydrolysis. Acidification of vesicles by intracellular V-ATPases is important for various cellular processes, including receptor-mediated endocytosis, membrane trafficking, and protein processing and degradation. They also function on the plasma membrane of certain cells, such as tumor cells, renal intercalated cells, and osteoclasts. Aberrant function of V-ATPase is associated with a number of human diseases including tumor metastasis, distal renal tubular acidosis, and osteoporosis. Therefore, they are considered as potential drug targets [5, 7]. V-ATPases are found in some bacteria, such as *Thermus thermophilus* (*T. thermophilus*) and *Enterococcus hirae* (*E. hirae*). V-ATPase from *T*. *thermophilus* functions as an ATP synthase under physiological conditions [8, 9]. Therefore, this enzyme is sometimes called A-type ATPase found in Archeae (A-ATPase). A-ATPases function as ATP synthases similar to the F-type ATP synthase, although the structure and subunit composition of A-ATPases are more similar to those of V-ATPases (Fig. 1a) [10–12]. V-ATPase from *E. hirae* functions as a sodium ion pump similar in nature to eukaryotic V-ATPase, and plays an important role in maintaining sodium homeostasis in cells under an alkaline environment [13–15].

All rotary ATPases are unique rotary molecular motors that function as energy conversion machines with a similar architecture and rotary catalytic mechanism [5, 11, 12, 16]. They are large, multi-subunit complexes composed of a hydrophilic F_1_/V_1_/A_1_ motor for ATP synthesis/hydrolysis and a membrane-embedded F_o_/V_o_/A_o_ motor for ion transport. The bacterial F_1_/V_1_/A_1_ and F_o_/V_o_/A_o_ motors are connected by one central stalk and one or two peripheral stalks (Fig. 1). Interestingly, V-ATPases in eukaryotes have three peripheral stalks, although there is only one peripheral stalk in eukaryotic F-ATPases [16–18]. The peripheral stalks of eukaryotic V-ATPases play an important role in reversible dissociation of V_1_ motor from V_o_ motor with silencing of the ATP hydrolysis activity of the free V_1_ motor [18], in contrast to F_1_ motor which will rapidly hydrolyze ATP when isolated. This implies the fact that it was necessary for V-ATPases to evolve a regulatory mechanism for keeping the dissociated V_1_ motors catalytically inactive to prevent wasteful energy consumption.

Among all known rotary ATPases, the hydrophilic portion of F-ATPase (F_1_-motor or F_1_-ATPase) is the best characterized; there are high-resolution crystal structures of several rotational states [19–28] and the chemomechanical coupling scheme has been revealed in detail by extensive single-molecule studies [29–40]. Relatively little is known about the structure and rotation scheme of V_1_-motors (V_1_-ATPase) [41–43]. Recently, high-resolution crystal structures of several rotational states of V_1_-ATPase from *E. hirae* have been determined [44, 45]. Furthermore, basic rotary dynamics of this V_1_-ATPase have been revealed by single-molecule studies [46–49]. These studies have provided new insights into the rotation mechanism of V_1_-ATPase. In the present review, we discuss recent findings on the structural features and rotary dynamics of bacterial V_1_-ATPase revealed from structural and single-molecule studies with a focus on differences from the properties of F_1_-ATPase.

## V_1_-ATPase: structural studies

**Fig. 2:**
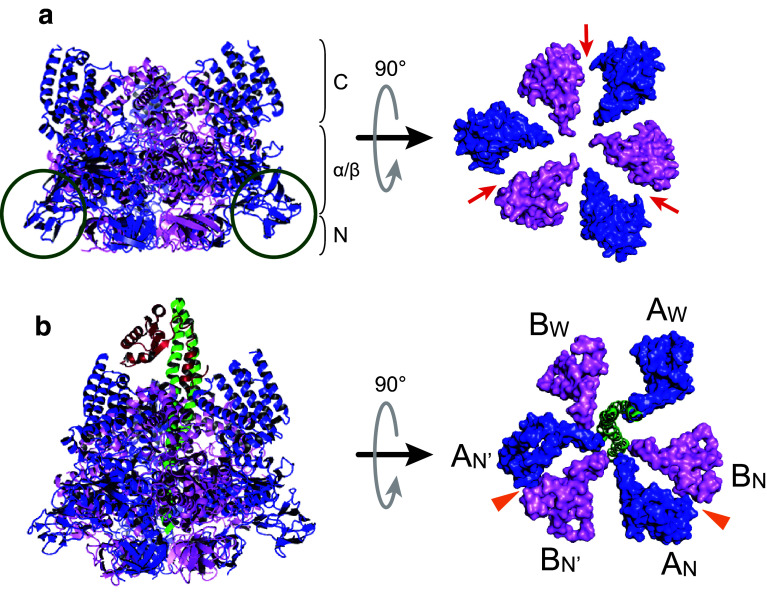
Crystal structures of A_3_B_3_ subcomplex and V_1_-ATPase from *T. thermophilus*. **a** Crystal structure of the nucleotide-free A_3_B_3_ subcomplex from *T. thermophilus* determined at 2.8 Å resolution (PDB ID: 3GQB) [41]. Side view (left) and Top view (right) from the membrane side. Only the C-terminal domains of the A_3_B_3_ ring are shown in top view to clarify the distinct conformations of individual A or B subunits. The A and B subunits are shown in blue and magenta, respectively. Each A and B subunit consists of the N-terminal β-barrel (N), the central α/β domain (α/β), and the C-terminal helical domain (C). Green circles indicate the “non-homologous region” in the A subunit. Red arrows indicate the catalytic sites. **b** Overall structure of V_1_-ATPase (A_3_B_3_DF) from *T. thermophilus* determined at 4.5 Å resolution (PDB ID: 3A5C) [42]. Side view (left) and top view (right) from the membrane side. Only the C-terminal domains of the A_3_B_3_ ring and the D subunit are shown in top view. The A, B, D, and F subunits are shown in blue, magenta, green, and red, respectively. A_W_, B_W_ pair shows a wide-open conformation, as observed in a α_E_β_E_ pair in F_1_-ATPase [19]. A_N_B_N_ and A_N′_B_N′_ pairs show a narrowly closed conformation, as do the α_TP_β_TP_ and α_DP_β_DP_ pairs in F_1_-ATPase [19]. The crystals were obtained by co-crystallization with Mg^2+^ ADP and aluminium fluoride. Strong electron densities which presumably correspond to the phosphate groups of bound-ADP were found in the A_N_B_N_ and A_N’_B_N’_ pairs (orange arrowheads)

In the bacterial V-ATPase, the catalytic V_1_ moiety is composed of A, B, D, and F subunits, in which three alternately arranged A and B subunits form a hexameric stator A_3_B_3_ ring. The central rotary shaft of D and F subunits penetrates the central cavity of the A_3_B_3_ ring and rotates using the energy of ATP hydrolysis [41–44]. Unlike the isolated eukaryotic V_1_ moiety in which subunit H inhibits its activity [18], the isolated bacterial V_1_ moiety can generally catalyze ATP hydrolysis and hence is called V_1_-ATPase. Structural studies have been conducted using a V_1_-ATPase from the thermophilic eubacterium *T*. *thermophilus*, which has high stability. The crystal structure of the A_3_B_3_ subcomplex from *T*. *thermophilus* was determined at 2.8 Å resolution [41]. The diameter of the A_3_B_3_ subcomplex is larger than that of the α_3_β_3_ subcomplex in F_1_-ATPase, because it includes an outward protrusion domain in the A subunit (Fig. 2a, green circles), termed the “non-homologous region” that is absent from β subunit; the structure also provides molecular information about the B–A interface. The catalytic sites are located at the interfaces of the A and B subunits (Fig. 2a, red arrows), with the majority of the catalytic residues residing in the A subunits, similar to the catalytic β subunits and the non-catalytic α subunits in F_1_-ATPase [19, 27]. The overall structure of V_1_-ATPase (A_3_B_3_DF complex) from *T*. *thermophilus* was first determined at 4.5–4.8 Å resolution (Fig. 2b) [42]. This structure provided the initial information about the position and orientation of the rotor DF subunits in the A_3_B_3_ ring and revealed structural similarities and differences between V_1_- and F_1_-ATPase. However, the lack of high-resolution structural information for the overall V_1_-ATPase from *T*. *thermophilus* limits our understanding of its molecular architecture and operation. Meanwhile, high-resolution crystal structures of the A_3_B_3_ ring and entire V_1_-ATPase from *E. hirae* have recently been solved with and without bound nucleotides [44, 45].

## Structure of A_3_B_3_ ring from *E. hirae*: asymmetric structure

**Fig. 3:**
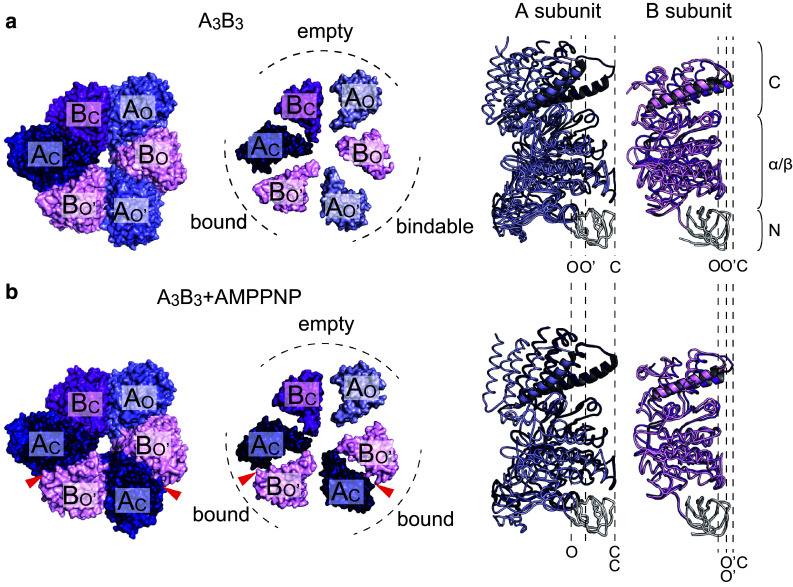
Crystal structures of A_3_B_3_ subcomplex from *E. hirae*. **a** Crystal structure of the nucleotide-free A_3_B_3_ subcomplex from *E. hirae* determined at 2.8 Å resolution (PDB ID: 3VR2) [44]. Each A and B subunit consists of the N-terminal β-barrel (N), the central α/β domain (α/β), and the C-terminal helical domain (C) as seen in A_3_B_3_ from *T. thermophilus* (Fig. 2a). **b** Crystal structure of the nucleotide-bound A_3_B_3_ subcomplex from *E. hirae* determined at 3.4 Å resolution (PDB ID: 3VR3) [44]. Two AMPPNP molecules are bound to the ‘bound’ sites (indicated by red arrowheads). Top views from the membrane side are shown on the left and center. Only the C-terminal domains of the A_3_B_3_ rings are shown in the center to clarify the distinct conformations of individual A or B subunits and the different structures of the three catalytic sites. On the right, conformations of the individual A and B subunits superimposed at the N-terminal β-barrel domain (white) are shown. *O and O′* open conformation, *C* closed conformation

The high-resolution crystal structures of the A_3_B_3_ ring from *E. hirae* were solved with and without a non-hydrolyzable ATP analog [adenosine 5′-(β,γ-imido)triphosphate or AMPPNP] at 3.4 and 2.8 Å resolution, respectively (Fig. 3) [44]. The overall architecture of A_3_B_3_ from *E. hirae* is similar to that of α_3_β_3_ in F_1_-ATPase, but the structures show some differences.

Each A and B subunit consists of an N-terminal β-barrel, central α/β domain, and C-terminal helical domain (Fig. 3a, right). Superimposition of the N-terminal β-barrel part of three A or B subunits shows that the conformations of each A and B subunit are not identical. If one of the A subunits is in the closed conformation (C), the other two A subunits show open conformations (O or O′). Similarly, one of the B subunits takes the closed form (C) and the other two take open conformations (O or O′) (Fig. 3a), resulting in the asymmetry of the A_3_B_3_ ring. Interestingly, even in the absence of nucleotides and central rotor DF subunits, each catalytic site shows the three distinct states. In contrast, F_1_-ATPase shows a threefold symmetric structure with three identical catalytic sites in the absence of bound nucleotides [50, 51]; accordingly, the asymmetric structure of the stator ring is not observed in the case of F_1_-ATPase. In the presence of high concentration of AMPPNP (5 mM), the A_3_B_3_ ring binds two AMPPNP molecules (Fig. 3b), resulting in changes in the conformation of A (O′ to C) and B (O to O′) subunits, but the A_C_B_O′_ pair shows little conformational change upon AMPPNP binding. Therefore, the three catalytic sites (A_O_B_C_, A_O′_B_O_, and A_C_B_O′_) are termed ‘empty’, ‘bindable’, and ‘bound’ sites, respectively (Fig. 3a, b).

## Two AMPPNP-bound structure of V_1_-ATPase: catalytic dwell state

**Fig. 4:**
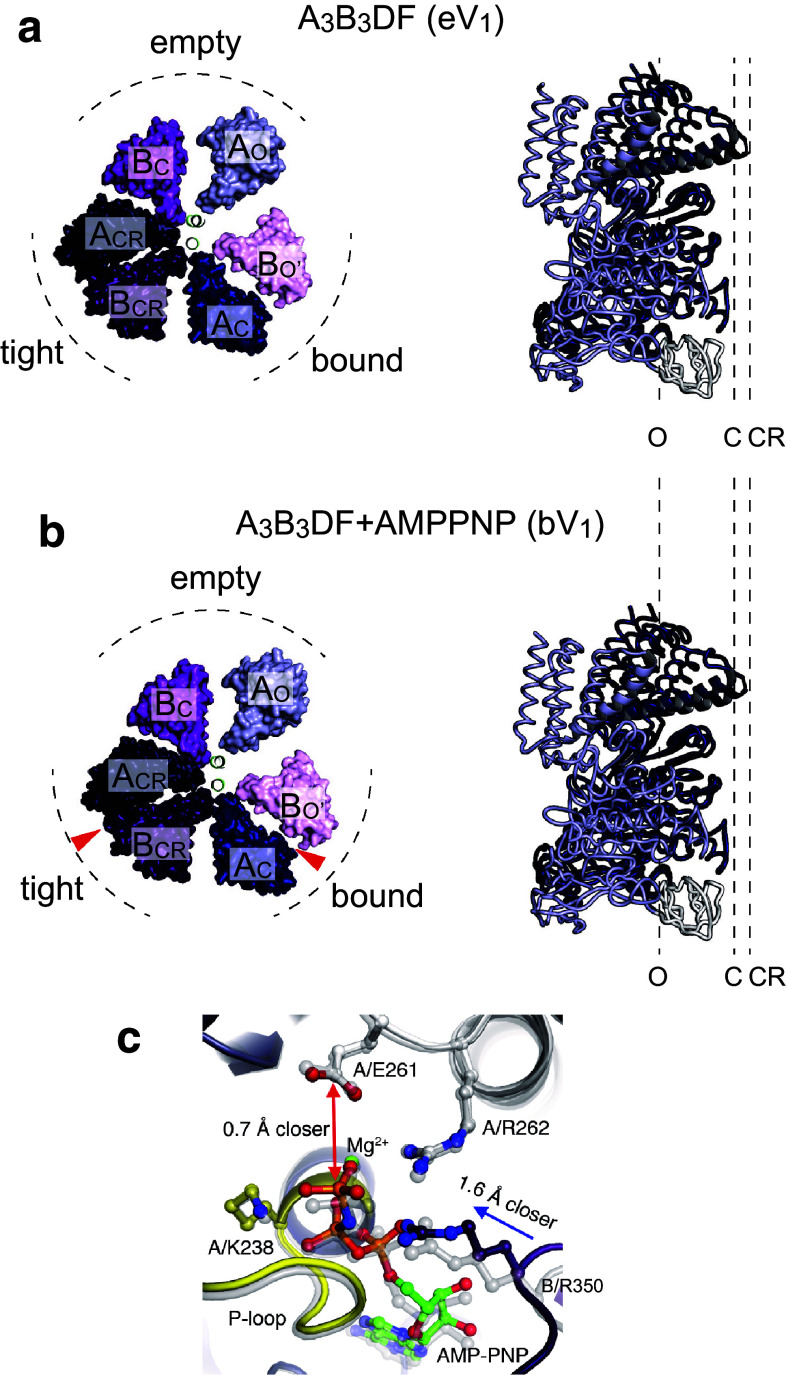
Crystal structures of entire V_1_-ATPase from *E. hirae*. **a** Crystal structure of the nucleotide-free V_1_-ATPase from *E. hirae* determined at 2.2 Å resolution (PDB ID: 3VR4) [44]. **b** Crystal structure of the nucleotide-bound V_1_-ATPase from *E. hirae* determined at 2.7 Å resolution (bV_1_, PDB ID: 3VR6) [44]. Two AMPPNP molecules are bound to the ‘bound’ and the ‘tight’ sites (indicated by red arrowheads). Top views from the membrane side are shown on the left. Only the C-terminal domains of the A_3_B_3_ rings and the α-helical coiled-coil portion of the D subunit are shown. On the right, conformations of the individual A subunit superimposed at the N-terminal β-barrel domain (white) are shown. *O* open conformation, *C* closed conformation, *CR* more closed ‘closer’ conformation. Two AMPPNP-bound V_1_-ATPase shows an almost identical structure to the nucleotide-free V_1_-ATPase. **c** Nucleotide-binding site of the bV_1_ (PDB ID: 3VR6). Superposition of the ‘bound’ site (transparent grey) and the ‘tight’ site (colored) of the bV_1_

**Fig. 5:**
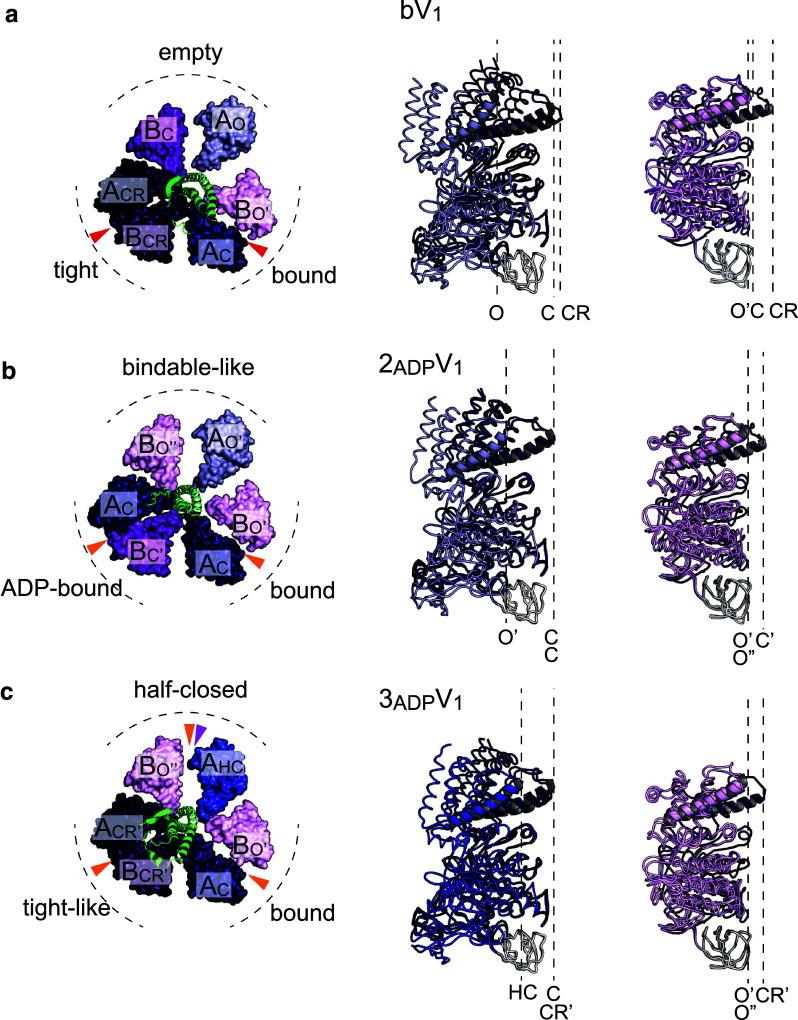
Crystal structures of two AMPPNP-bound V_1_-ATPase (bV_1_), two ADP-bound V_1_-ATPase (2_ADP_V_1_), and three ADP-bound V_1_-ATPase (3_ADP_V_1_) from *E. hirae*. **a** Crystal structure of two AMPPNP-bound V_1_-ATPase (bV_1_, 2.7 Å resolution, PDB ID: 3VR6) [44]. **b** Crystal structure of two ADP-bound V_1_-ATPase (2_ADP_V_1_, 3.3 Å resolution, PDB ID: 5KNB) [45]. **c** Crystal structure of three ADP-bound V_1_-ATPase (3_ADP_V_1_, 3.0 Å resolution, PDB ID: 5KNC) [45]. Top views of the C-terminal domain of A_3_B_3_ rings and central rotor D subunit (green) viewed from the membrane side are shown on the left. Red, orange, and magenta arrowheads indicate the catalytic sites that bind to AMPPNP, ADP, and sulfate, respectively. Conformations of the individual A and B subunits superimposed at the N-terminal β-barrel domain (white) are shown on the right. *O and O′* open conformation, *HC* half-closed conformation, *C* closed conformation, *CR* more closed ‘closer’ conformation

In addition to the A_3_B_3_ ring structures, the nucleotide-free entire V_1_-ATPase from *E. hirae* (eV_1_) was also determined at 2.2 Å resolution [44]. Insertion of the rotor DF subunits into the stator A_3_B_3_ ring induces conformational changes in the A and B subunits, even in the absence of bound nucleotides (Fig. 4a). This results in changes in the conformations of A and B subunits from the closed (C) to the more closed ‘closer’ (CR) conformation and from the open (O') to closer (CR) conformation, respectively. Consequently, eV_1_ shows three different catalytic sites termed ‘empty’, ‘bound’, and ‘tight’ sites (Fig. 4a). Furthermore, by soaking eV_1_ in AMPPNP, the structure of nucleotide-bound V_1_-ATPase (bV_1_ or 2_ATP_V_1_) was also determined at 2.7 Å resolution (Fig. 4b) [44, 45]. Two AMPPNP molecules were bound to the binding sites of the ‘bound’ and ‘tight’ sites of eV_1_, but the overall structure was very similar to that of eV_1_ (Fig. 4a, b). Even in the presence of high AMPPNP (2 mM), no electron density peak for AMPPNP was found in the ‘empty’ site, indicating that it has a very low affinity for AMPPNP. Comparison of the ‘tight’ and ‘bound’ sites in bV_1_ revealed the movement of the Arg-finger (Arg-350) in the ‘tight’ site closer to the γ-phosphate relative to the ‘bound’ site (Fig. 4c). This γ-phosphate moved closer to Glu-261 in the A subunit which is essential for ATPase activity in yeast V_1_-ATPase [52]. The corresponding Glu-188 of the β subunit in bovine mitochondrial F_1_-ATPase is an essential residue for ATP hydrolysis and interacts with the γ-phosphate of the nucleotide and lytic water molecules [19, 28, 53]. The closer proximity of the Arg-finger to the γ-phosphate may enhance the ATP hydrolysis reaction. Therefore, it is possible that the ‘tight’ site corresponds to the catalytic site waiting for ATP hydrolysis and this structure corresponds to the catalytic dwell state (Fig. 5a). Similar nucleotide-free and nucleotide-bound structures of yeast mitochondrial F_1_-ATPase have been reported [25]. These results suggest that interactions between the rotor and stator are as crucial as nucleotide binding for determining the structure of the catalytic sites of rotary ATPases.

## Two ADP-bound structure of V_1_-ATPase: ATP-binding dwell state

More recently, the crystal structures of two other nucleotide-bound states were reported [45], i.e., the two ADP-bound structure (2_ADP_V_1_) and the three ADP-bound structure (3_ADP_V_1_). When soaking the eV_1_ crystals in 20 µM ADP, it binds to the ‘bound’ and ‘tight’ sites of eV_1_, as in the case of bV_1,_ and the two ADP-bound structure was solved at 3.3 Å resolution (Fig. 5b). ADP binding to the ‘tight’ site in eV_1_ induces changes in the A and B subunits to more open conformations (CR to C in A subunit; CR to C′ in B subunit), but the ‘bound’ site in eV_1_ shows no conformational change upon ADP binding. The observed conformational changes result in the tilting of rotor DF subunits towards the ADP-bound site (A_C_B_C′_) (Fig. 5b). Interestingly, the ‘empty’ site shows a cooperative conformational change without ADP binding. The newly found catalytic sites (A_C_B_C′_ and A_O′_B_O′′_) are termed ‘ADP-bound’ and ‘bindable-like’ sites, respectively. The ‘bindable-like’ site is similar to the ‘bindable’ site in the A_3_B_3_ structure (Fig. 3a) and takes a more open conformation than that of the ‘empty’ site. Therefore, the ‘bindable-like’ site is considered to be the catalytic site waiting for ATP binding, and the 2_ADP_V_1_ structure is regarded as the ATP-binding dwell state.

## Three ADP-bound structure of V_1_-ATPase: ADP-release dwell state

**Fig. 6:**
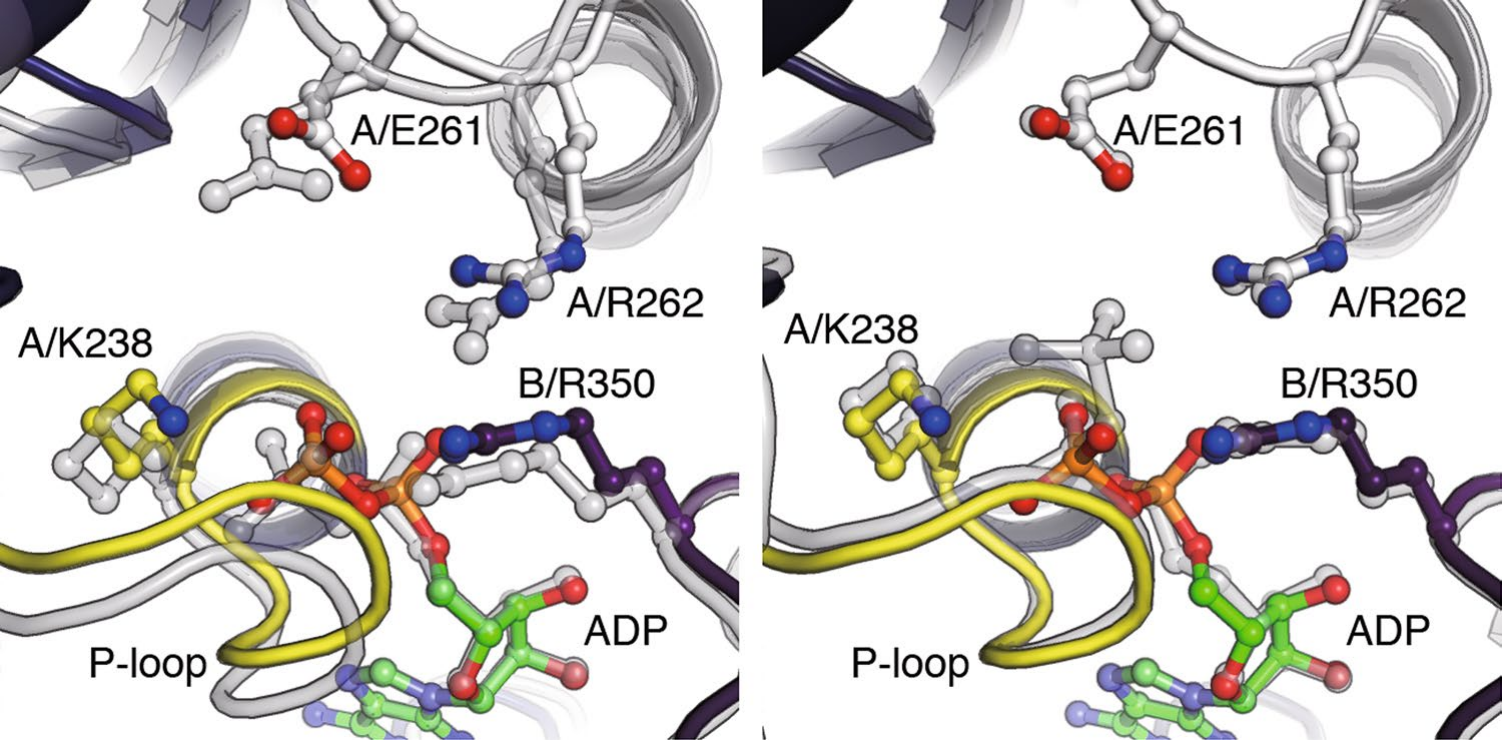
Nucleotide-binding site of the three ADP-bound V_1_-ATPase (3_ADP_V_1_). Nucleotide-binding site of the “tight-like” site in 3_ADP_V_1_ (colored) is superimposed at the adenosine onto those (transparent grey) of the “ADP-bound” site in 2_ADP_V_1_ (left) and the “tight” site in bV_1_ (right)

The 3_ADP_V_1_ structure was solved at 3.0 Å resolution by soaking the eV_1_ crystals in 2 mM ADP [45]. In this structure, all three catalytic sites are occupied by ADP and, in addition, a sulfate is bound to one catalytic site (Fig. 5c, magenta arrowhead). A comparison between 2_ADP_V_1_ and 3_ADP_V_1_ structures shows that ADP (and sulfate) binding to the ‘bindable-like’ site in 2_ADP_V_1_ induces a conformational change in the A subunit (O′) to the ‘half-closed’ conformation (HC), whereas the B subunit (O′′) shows no conformational change. This catalytic site (A_HC_B_O′′_) in 3_ADP_V_1_ is termed a ‘half-closed’ site. The conformational change in the catalytic site from the ‘bindable-like’ site to the ‘half-closed’ site induces a conformational change of the ‘ADP-bound’ site to a more tight-like conformation (A_CR′_B_CR′_). This shifted ‘ADP-bound’ site in 3_ADP_V_1_ is termed a ‘tight-like’ site, because the nucleotide-binding site is more similar to that of the ‘tight’ site than to that of the ‘ADP-bound’ site (Fig. 6). The β-phosphate of ADP in the ‘tight-like’ site is more distant from the surrounding interacting residues compared to that in the ‘ADP-bound’ site, suggesting that an ADP will be easily released from the ‘tight-like’ site (Fig. 6) [45]. Therefore, the “tight-like” site is considered to be a dwelling state before ADP release and the 3_ADP_V_1_ structure is regarded as the ADP-release dwell state.

## Structural difference between F_1_-and V_1_-ATPase: conformational features

**Fig. 7:**
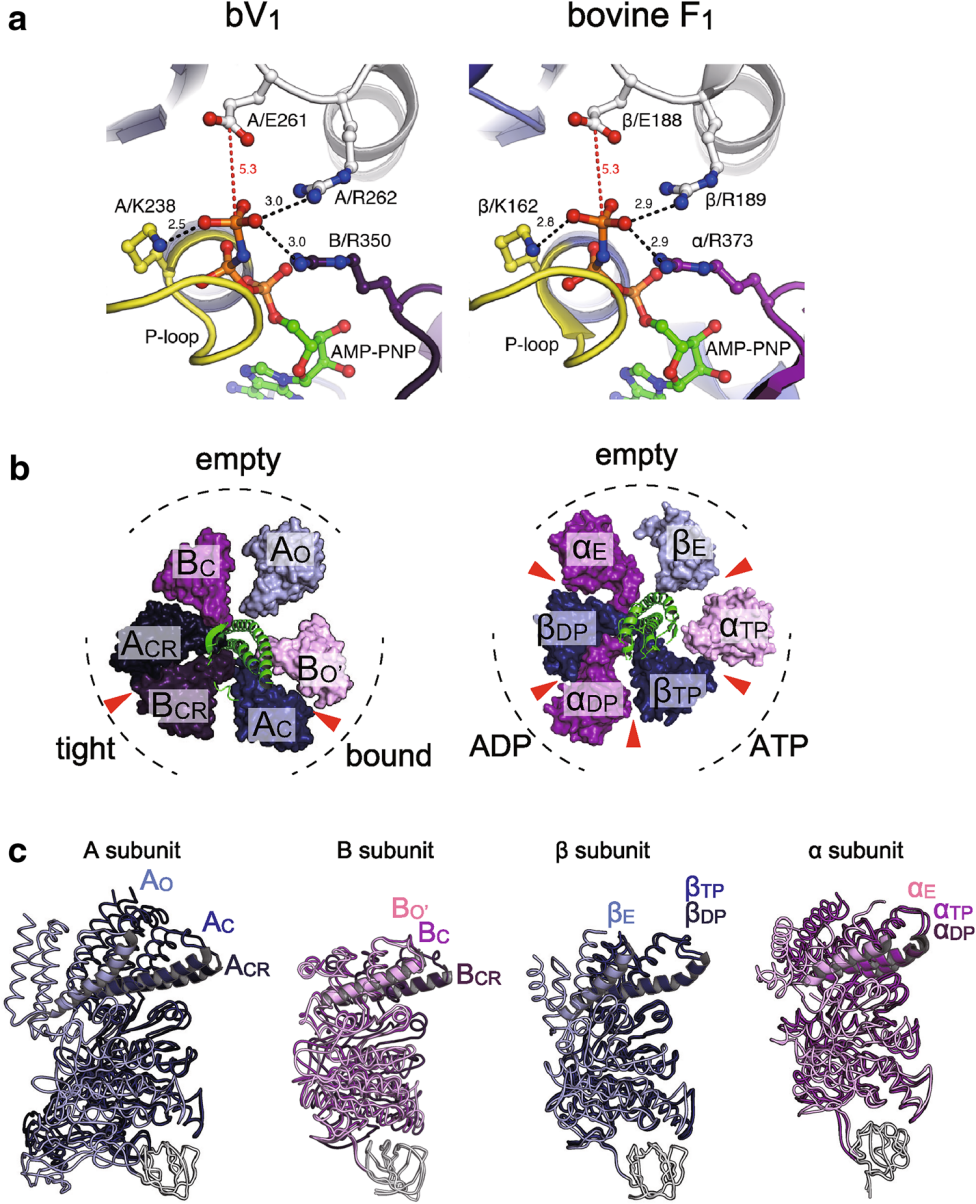
Structural differences between F_1_-and V_1_-ATPase. **a** Comparison of the nucleotide-binding site of the “tight” site in *E. hirae* V_1_-ATPase (bV_1_, PDB ID: 3VR6) with that of the “ADP-bound” site in bovine F_1_-ATPase (PDB ID: 2JDI) [28]. **b** Top views from the membrane side of bV_1_ (left) and bovine F_1_-ATPase (right). The catalytic and non-catalytic sites that bind to AMPPNP molecules are indicated by red arrowheads. **c** Superimposed structures at the N-terminal β-barrel (white) of three structures of A and B subunits in bV_1_ compared with the β and α subunits in F_1_-ATPase

**Fig. 8:**
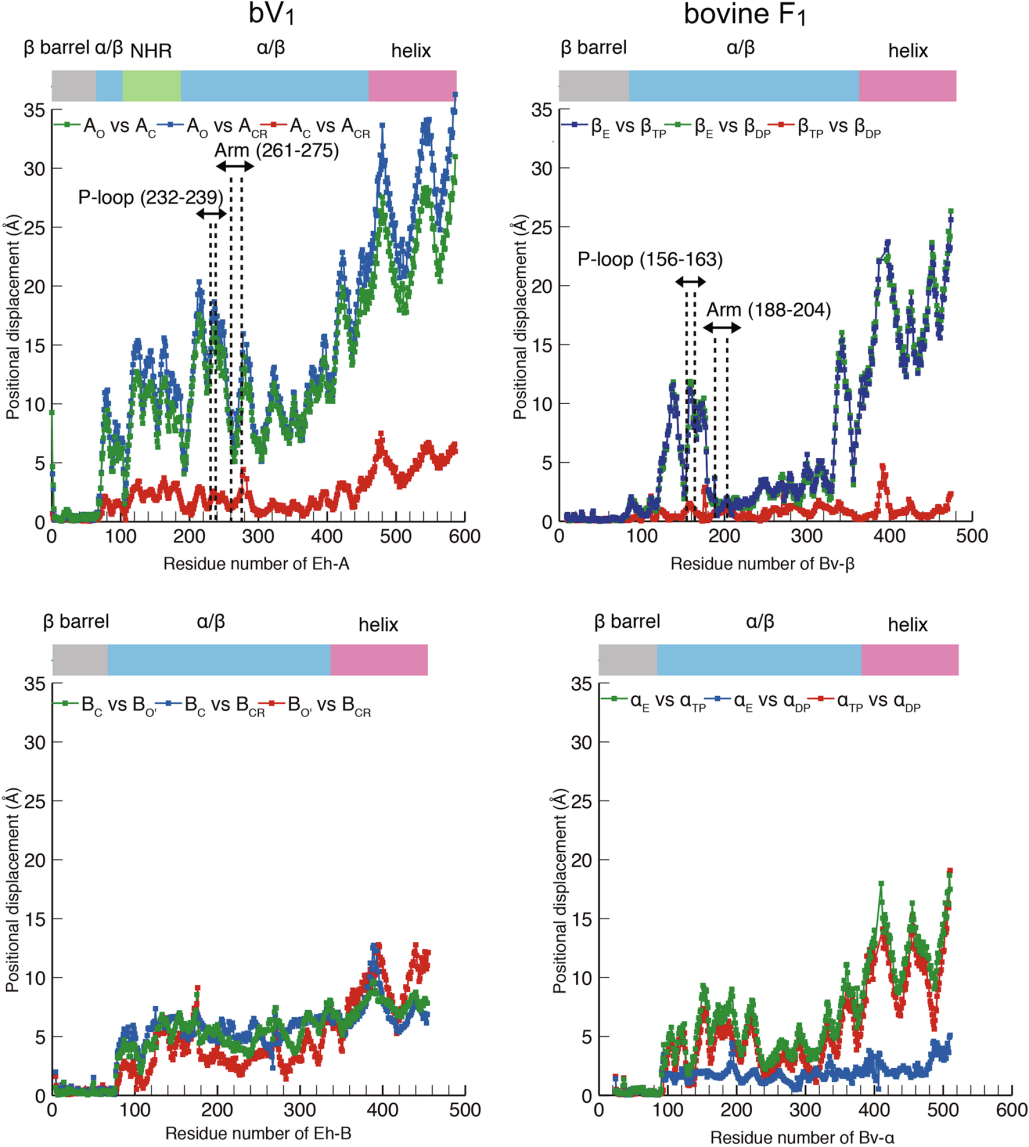
Comparison of the conformational differences between F_1_- and V_1_-ATPase. Positional displacement of residues (Cα atoms) between two of the three A subunits (top left) and B subunits (bottom left) in bV_1_, and β subunits (top right) and α subunits (bottom right) in bovine F_1_-ATPase (PDB ID: 2JDI), which are superimposed at the N-terminal β-barrel domains (see Fig. 7c)

A comparison of the two AMPPNP-bound structures of V_1_-ATPase from *E. hirae* [44, 45] and F_1_-ATPase from bovine mitochondria [28] is shown in Fig. 7. Both nucleotide-binding sites show very similar arrangements of catalytically important residues and nucleotides (Fig. 7a). However, the overall structures of A and B subunits in V_1_-ATPase show some differences from those of the β and α subunits in F_1_-ATPase. The non-catalytic B subunit of this V_1_-ATPase does not bind to a nucleotide, whereas the non-catalytic α subunit of F_1_-ATPase binds to a nucleotide (Fig. 7b). Superimposition of the N-terminal β-barrel part of three A and B subunits in V_1_-ATPase and β and α subunits in F_1_-ATPase reveals conformational differences between A_C_ and A_CR_, and B_C_ and B_CR_ of V_1_-ATPase, but the conformations of β_TP_ and β_DP_ as well as α_TP_ and α_DP_ of F_1_-ATPase are very similar (Fig. 7c). These differences are also evidenced by the positional displacement of residues between two of the three A and B subunits in V_1_-ATPase and β and α subunits in F_1_-ATPase (Fig. 8), which also shows that the structures of A_C_ and A_CR_ are largely different from that of A_O_ (Fig. 8, top left). In contrast, the central portions (residues 180–320) of the β_TP_ and β_DP_ structures show very similar conformations to that of the β_E_ structure (Fig. 8, top right). These results suggest that the A subunit in V_1_-ATPase from *E. hirae* undergoes the whole conformational change upon nucleotide binding, whereas the β subunit in F_1_-ATPase undergoes the conformational change mainly in the P-loop and C-terminal domains.

Thus, the conformational features of the A and B subunits in V_1_-ATPase from *E. hirae* are apparently different from those of the β and α subunits in bovine F_1_-ATPase, despite the highly similar nucleotide-binding sites of these ATPases. These structural differences and similarities may be related to the ‘unique’ and ‘common’ mechanisms of rotary catalysis between these rotary ATPases, such as the chemomechanical coupling scheme of the rotation [40, 46].

## Dynamics of rotary V_1_-ATPase: single-molecule studies

After the establishment of the single-molecule rotation assay of F_1_-ATPase in 1997 [29], the dynamics of rotary ATPases from various species have been studied at the single-molecule level [38–40, 46, 54–57]. The first rotation of V_1_-ATPase has been directly visualized under an optical microscope by the attachment of large beads to the rotor DF subunits in V_1_-ATPase from *T. thermophilus* [58]. V_1_-ATPase from *T. thermophilus* rotates stepwise in a counterclockwise direction, consuming one ATP molecule at each step when viewed from the membrane side. The basic step size is 120°, which is similar to that of F_1_-ATPase, and no substeps have been resolved in the rotation, even when using a slowly hydrolyzable ATP analog (adenosine 5′-*O*-(3-thio)triphosphate or ATPγS) and high-speed imaging of gold nanoparticles [48, 59]. These results indicate that ATP binding and ATP cleavage (and/or phosphate release) occur at the same angle in this V_1_-ATPase. In the case of F_1_-ATPases, some or all of these elementary reaction steps occur at different angles and the basic 120° step is further divided into two or three substeps [31, 32, 39, 40], i.e., 80° and 40° substeps in thermophilic *Bacillus* PS3 F_1_-ATPase [31, 32], 85° and 35° substeps in *Escherichia coli* F_1_-ATPase [39], and 65°, 25°, and 30° substeps in human F_1_-ATPase [40]. The 80°, 85°, and 65° substeps are triggered by ATP binding and ADP release, while the 40° and 35° substeps are triggered by ATP cleavage and phosphate release. In human F_1_-ATPase, it is proposed that ATP cleavage and phosphate release trigger different substeps of 30° and 25°, respectively.

**Fig. 9:**
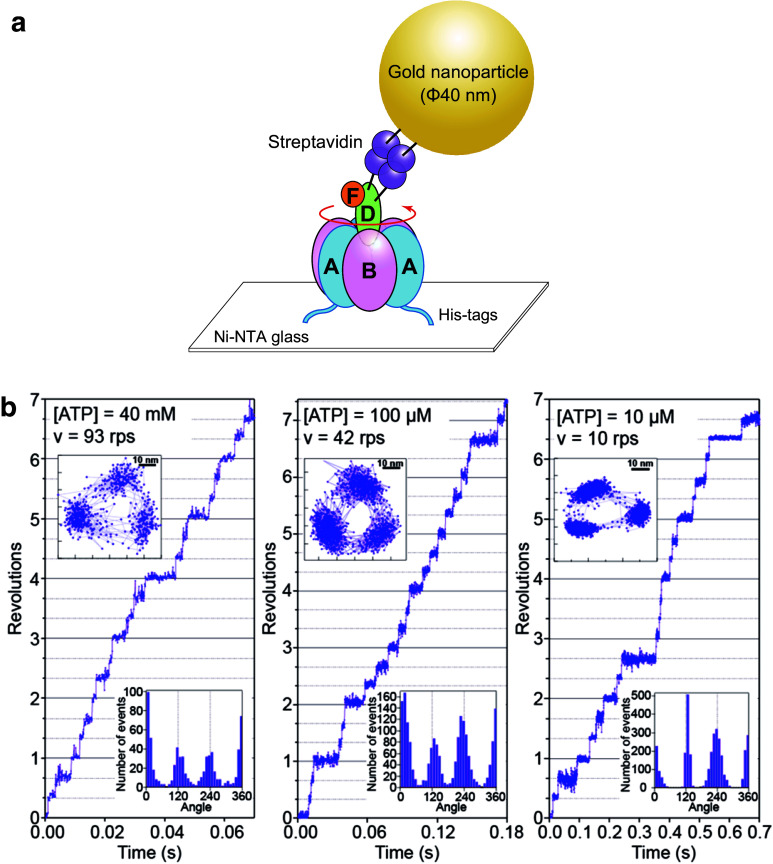
Single-molecule rotation of *E. hirae* V_1_-ATPase. **a** Schematic illustration of the single-molecule rotation assay of *E. hirae* V_1_-ATPase. The A_3_B_3_ ring is immobilized on a glass surface via a His-tag on the A subunit, and an optical probe (gold nanoparticle, 40 nm in diameter) is attached to the D subunit to visualize the rotary motion of rotor DF subunits using an optical microscope [46, 68]. **b** Rotations of *E. hirae* V_1_-ATPase for various concentrations of ATP. Left: 40 mM ATP, considerably higher than the Michaelis constant (*K*_m_, 154 µM). Center: 100 µM ATP, near the *K*_m_. Right: 10 mM ATP, considerably lower than the *K*_m_ [46]

Recently, rotary dynamics of V_1_-ATPase from *E. hirae* have been characterized using single-molecule analyses at a submillisecond temporal resolution employing gold nanoparticles and an objective-type total internal reflection dark field microscope (Fig. 9a) [46–48]. V_1_-ATPase from *E. hirae* rotates in basically the same manner as that from *T. thermophilus* which functions as ATP synthase [55, 59]. This V_1_-ATPase also shows only three pauses separated by 120° at all concentrations ranging from below to above the Michaelis constant (*K*_m_), where distinct elementary reaction steps of ATP hydrolysis (ATP binding, ATP cleavage, or product release) become the rate-limiting step (Fig. 9b), suggesting that 120° stepping rotation without substeps is a common property of V_1_-ATPase [46], despite the difference in physiological function between V-ATPase from *E. hirae* (ion pump) and that from *T. thermophilus* (ATP synthesis). These results imply that the basic properties of rotary dynamics of F-ATPases and V-ATPases are determined by their overall structures and that the difference in physiological function derives from regulatory mechanisms such as Mg^2+^ ADP inhibition and inhibitor proteins [9, 18, 23, 26].

## Chemomechanical coupling scheme of V_1_-ATPase

**Fig. 10:**
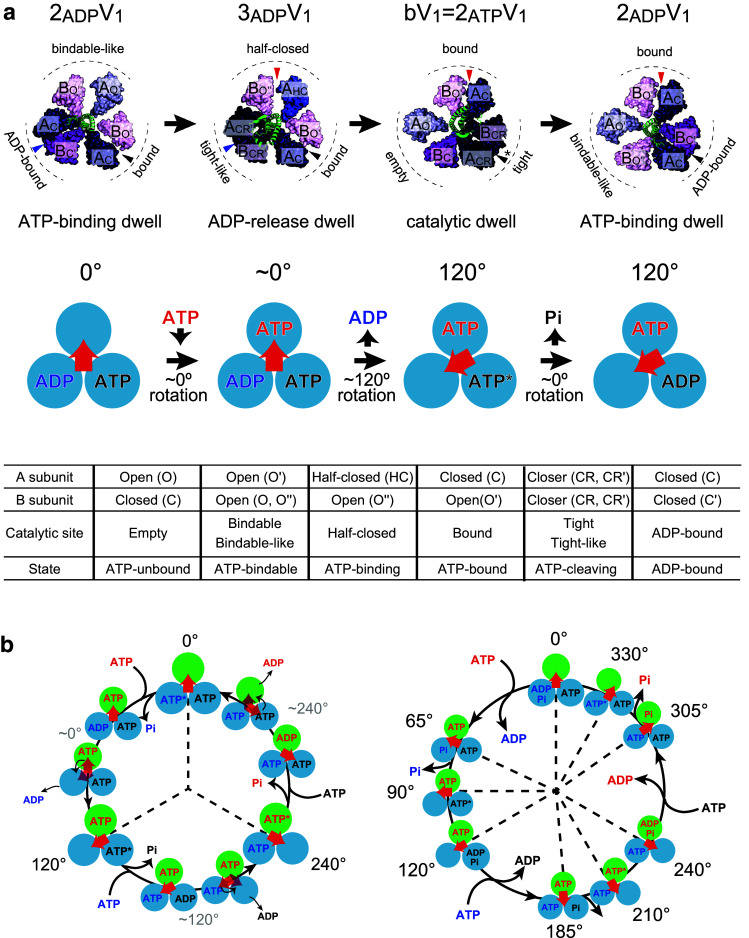
Chemomechanical coupling scheme of V_1_-ATPase. **a** Proposed rotation model of *E. hirae* V_1_-ATPase for 120° rotation [45]. The structure models are based on the crystal structures of 2_ADP_V_1_ (ATP-binding dwell), 3_ADP_V_1_ (ADP-release dwell), and bV_1_ (catalytic dwell). The catalytic sites that bind to nucleotides are indicated by arrowheads (top). Each blue circle represents the chemical state of each catalytic site, and the central red arrow represents the orientation of the rotor DF subunits. ATP* represents the pre- or post-hydrolysis state of ATP (middle). Correspondence table for all catalytic sites observed in the crystal structures of the A_3_B_3_ and V_1_-ATPase (bottom). **b** Possible chemomechanical coupling scheme of *E. hirae* V_1_-ATPase [44–46, 48, 49] (left) and human F_1_-ATPase [40] (right) for 360° rotation. 0° is set as the ATP-binding angle for the catalytic site at the 12 O’clock position (green). In the model of *E. hirae* V_1_-ATPase, ATP bound at 0° is cleaved into ADP and Pi at 240°. Among these, phosphate first dissociates at 240°, and then, ADP release occurs at 240°. Other catalytic sites also obey the same reaction scheme offset by 120° and 240°. In the model of human F_1_-ATPase, ATP bound at 0° is cleaved into ADP and Pi at 210°, ADP dissociates at 240°, and then, phosphate release occurs at 305°

Figure 10a shows a proposed chemomechanical coupling scheme of V_1_-ATPase from *E. hirae* based on recently determined structural features and rotary dynamics [44–47]. As mentioned above, three distinct structures of *E. hirae* V_1_-ATPase have been solved (bV_1_ = 2_ATP_V_1_, 2_ADP_V_1_, and 3_ADP_V_1_) [44, 45]. These three structures are regarded as the three different dwelling states in the rotation waiting for the elementary reaction steps of ATP hydrolysis corresponding, respectively, to the catalytic (ATP cleavage) dwell (bV_1_ = 2_ATP_V_1_), ATP-binding dwell (2_ADP_V_1_), and ADP-release dwell (3_ADP_V_1_) states.

In this model, ATP binds to the ‘bindable-like’ site in 2_ADP_V_1_, which induces conformational changes of ‘bindable-like’ and ‘ADP-bound’ sites to ‘half-closed’ and ‘tight-like’ sites, respectively. This results in a slight shift of the rotor DF subunits toward the ‘tight-like’ site, but does not induce the obvious rotation of the rotor DF subunits. Then, ADP release occurs at the ‘tight-like’ site in 3_ADP_V_1_, and all three catalytic sites change the conformations from ‘half-closed’ to ‘bound’, from ‘bound’ to ‘tight’, and from ‘tight-like’ to ‘empty’. Coupled with these conformational changes, the DF subunits rotate 120°. After this rotation, ATP bound at the ‘tight’ site in bV_1_ (= 2_ATP_V_1_) is cleaved into ADP and phosphate. The release of phosphate, which has a lower affinity than ADP, is coupled with a conformational change from a ‘tight’ site to ‘ADP-bound’ site and from an ‘empty’ site to ‘bindable-like’ site. Consequently, the rotor DF subunits tilt toward the ‘ADP-bound’ site, without showing obvious rotation. Finally, it returns to the initial rotational state with 120° rotation (Fig. 10a).

Currently, there are no single-molecule studies on V_1_-ATPase from *E. hirae* that directly demonstrate the dwell angles for ATP cleavage, ADP release, and phosphate release at a single catalytic site over one revolution. However, considering the results of single-molecule studies and structural studies, the model for 360° rotation cycle is conceivable [48].

If the ATP-binding angle is defined as 0° in the 360° rotation cycle (Fig. 10b, left), ATP is cleaved at 240°. Then, phosphate is released first at 240°, and finally, ADP is released at 240°. In comparison, the proposed chemomechanical coupling scheme of F_1_-ATPase is more complicated. In the case of thermophilic *Bacillus* PS3 F_1_-ATPase, ATP cleavage, ADP release, and phosphate release occur at 200°, 240°, and 320° [37], respectively, although the timing of phosphate release is controversial. Furthermore, in the case of human F_1_-ATPase, ATP cleavage, ADP release, and phosphate release occur at 210°, 240°, and 305°, respectively (Fig. 10b, right) [40]. The coupling scheme of human F_1_-ATPase can be considered a variation of that of thermophilic *Bacillus* PS3 F_1_-ATPase, in which the ATP cleavage and the phosphate-release dwells are split into different angles. Of course, it is possible that V_1_-ATPase also shows the substeps, because the angles waiting for ADP release and phosphate release have not been directly demonstrated by advanced single-molecule experiments, as has been performed in the case of F_1_-ATPase [34, 35, 37]. Furthermore, multiscale molecular dynamics simulations predict the formation of rotational intermediate states of this V_1_-ATPase that have not yet been resolved [60]. Interestingly, the recent information-based soft clustering method revealed that thermophilic *Bacillus* PS3 F_1_-ATPase makes a small rotational movement during the catalytic dwell triggered by the ATP hydrolysis reaction; this had not been previously resolved using the conventional analysis methods [61]. Such advanced single-molecule techniques and analysis methods may reveal the unresolved reaction scheme and movements of V_1_-ATPase.

## Future prospects

The recent high-resolution structural studies and single-molecule studies reviewed have begun to clarify the rotation mechanism of V_1_-ATPase. By comparing the differences and similarities in the rotation mechanism between V_1_-ATPase and F_1_-ATPase, we can determine the ‘unique’ and ‘common’ mechanisms by which these rotary ATPases function and thereby establish the working principle of rotary ATPases. However, to fully understand the rotation mechanism of rotary ATPases, it is necessary to further improve our ‘knowledge and understanding’ by designing rotary ATPases with improved, modified, or novel functions based on our current ‘knowledge and understanding,’ and by experimentally verifying designed proteins. Such a synthetic approach has become a trend in biology and nanobiotechnology [62–67], and this approach will be extremely helpful to understand the mechanisms by which rotary ATPases operate.

## Abbreviations

AMPPNP: Adenosine 5′-(β,γ-imido)triphosphate
ATPγS: Adenosine 5′-*O*-(3-thio)triphosphate
